# Jaw position uncertainty and adjacent fields in breast cancer radiotherapy

**DOI:** 10.1120/jacmp.v16i6.5673

**Published:** 2015-11-08

**Authors:** Emma Hedin, Anna Bäck, Roumiana Chakarova

**Affiliations:** ^1^ Department of Radiation Physics Institute of Clinical Sciences, Sahlgrenska Academy, University of Gothenburg Gothenburg Sweden; ^2^ Department of Medical Physics and Biomedical Engineering Sahlgrenska University Hospital Gothenburg Sweden

**Keywords:** breast cancer, locoregional treatment, adjacent fields, dose distribution, Monte Carlo

## Abstract

Locoregional treatment of breast cancer involves adjacent, half blocked fields matched at isocenter. The objective of this work is to study the dosimetric effects of the uncertainties in jaw positioning for such a case, and how a treatment planning protocol including adjacent field overlap of 1 mm affects the dose distribution. A representative treatment plan, involving 6 and 15 photon beams, for a patient treated at our hospital is chosen. Monte Carlo method (EGSnrc/BEAMnrc) is used to simulate the treatment. Uncertainties in jaw positioning of ±1 mm are addressed, which implies extremes in reality of 2 mm field gap/overlap when planning adjacent fields without overlap and 1 mm gap or 3 mm overlap for a planning protocol with 1 mm overlap. Dosimetric parameters for PTV, lung and body are analyzed. Treatment planning protocol with 1 mm overlap of the adjacent fields does not considerably counteract possible underdosage of the target in the case studied. ${\text{PTV}‐V}_{95\%}$ is for example reduced from 95% for perfectly aligned fields to 90% and 91% for 2 mm and 1 mm gap, respectively. However, the risk of overdosage in PTV and in healthy soft tissue is increased when following the protocol with 1 mm overlap. A 3 mm overlap compared to 2 mm overlap results in an increase in maximum dose to PTV, ${\text{PTV}‐D}_{2\%}$, from 113% to 121%. $V_{120\%}$ for ‘Body‐PTV’ is also increased from $5\;{\text{cm}}^{3}$ to $14\;{\text{cm}}^{3}$. A treatment planning protocol with 1 mm overlap does not considerably improve the coverage of PTV in the case of erroneous jaw positions causing gap between fields, but increases the overdosage in PTV and doses to healthy tissue, in the case of overlapping fields, for the case investigated.

PACS numbers: 87.55.D‐, 87.55.dk, 87.55.Gh, 87.55.K‐, 87.56.J‐

## I. INTRODUCTION

Adjuvant radiotherapy after breast conserving surgery is used to reduce the risk for local recurrences. Regional lymph nodes are included (denoted as locoregional treatment) when lymph nodes show cancer involvement. In the locoregional case the target volume is treated in two parts, a cranial and a caudal one. The cranial part covers the lymph nodes and consists of anterior–posterior fields, whereas tangential fields are most commonly used for the caudal part (i.e., the breast tissue). The isocenter is positioned at the junction between the cranial and the caudal fields to eliminate beam divergence, which makes the treatment fields strongly asymmetrical (half‐blocked fields). This is a well‐established technique introduced many years ago,^(1)^ and is still used, also in combination with advanced respiratory gating systems.^(2)^

It is important to carefully align adjacent fields in order to maintain dose homogeneity in target without increasing dose to healthy tissue. The issue is closely related to the accuracy of the jaws positioning. Various methods for matchline dosimetry analysis have been documented.^3^, ^4^ Homann et al.^(3)^ reported a matchline dosimetry analysis tool based on irradiation of film in a phantom. Different field matching configurations for locoregional breast cancer treatment are studied in a plane in the abutment region and cold spots are detected. Madebo et al.^(4)^ investigated an implementation of EPID (electronic portal imaging device). A method based on EPID has been developed earlier in our hospital, where images of adjacent fields are analyzed for particular gantry angle. Jaw positional uncertainty of up to 1 mm has been detected for the Varian Clinac iX accelerators in our hospital, sometimes systematic shifts that holds during entire patient courses. The results from such quality control (QC) tests might be desirable to take into account in the treatment planning routines. For example, by planning adjacent fields with a certain overlap if the distance between the fields in the QC reveals gap. However, it is difficult to predict the absorbed dose that will be delivered in the junction region at the stage of planning since the technical tolerance in jaw position and in collimator rotation causing misalignment of the jaws results in unknown variations of the junction properties in each particular case. Treatment machines, regarded as identical, may be different in reality and present opposite behavior. The importance of alignment of adjacent fields grows with the implementation of more advanced systems for patient positioning and monitoring, when daily setup errors become smaller and no longer smoothen eventual dose differences between treatment fractions to the same degree. A field overlap of 1 mm is recommended in the planning protocol in our hospital to minimize the risk for underdosage in the target volume. This implies that the risk for hot spots is deliberately increased, since there is always a trade‐off between risk for hot spots and risk for cold spots. At the stage of planning, the 1 mm overlap is introduced and the matchline dose assumed to be adequate without further analysis (i.e., dosimetric effect of jaw positioning uncertainty is not evaluated for). The effect of the matching techniques on the dose variations in the junction region has been investigated in the case of a breast phantom within the large multicentre program START.^(5)^ Further dosimetric studies involving patient geometries are needed to quantify the dose inhomogeneity and evaluate clinical aspects related to it.

The objective of this work is to study the influence of the uncertainties in the jaw position on the dose distribution in the patient geometry of a locoregional breast cancer treatment and, furthermore, how a treatment planning protocol including field overlap of 1 mm affects the situation. This case study will contribute to the understanding of the benefits and disadvantages of using 1 mm overlap and if there is a need for further optimization of such a treatment protocol. The MC method is used to obtain the dose distributions. It is a reference method for validation of clinical dose calculations in the presence of heterogeneities, in the penumbra and in the buildup region and allows for a 3D dose evaluation including the use of dose‐volume histogram parameters currently used to specify dose planning criteria. The effect of ±1 mm uncertainty in the jaw positioning is investigated by the two extreme situations of gap and overlap of the adjacent fields that may happen in the reality. In particular, these extremes are 2 mm gap or overlap in the case of a planning protocol without gap or overlap, as well as 1 mm gap and 3 mm overlap in the case of a planning protocol with 1 mm overlap (used in our hospital for all locoregional breast cancer treatments).

## II. MATERIALS AND METHODS

Photon treatment fields from Varian Clinac iX accelerators (Varian Medical Systems, Palo Alto, CA) are considered. A MC model developed earlier,^6^, ^7^ built within EGSnrc/BEAMnrc code package,^8^, ^9^ is used for the calculations. The model is expanded for this study by including multileaf collimator (MLC) and dynamic wedges, as well as correction for backscatter to the monitor chamber, as described in the Appendix.

The capability of the 6 MV model to correctly reproduce asymmetric adjacent fields has been partly evaluated earlier.^(7)^ This evaluation is extended for the half‐blocked fields.

The traditional two‐steps approach is utilized, where the particles emerging from the accelerator head for a certain planned field are stored in a phase space file, which is further used as a source for dose calculations in the geometry of interest.

The Monte Carlo method is chosen to obtain results that are not dependent on a particular dose calculation algorithm currently available in a treatment planning system. However, test calculations are performed with the dose calculation algorithm currently used at our hospital for this type of treatment, namely the analytical anisotropic algorithm (AAA) version 10.0.28 implemented in Eclipse (Varian Medical Systems).

### A. Validation of MC beam calculations

**Figure 1:**
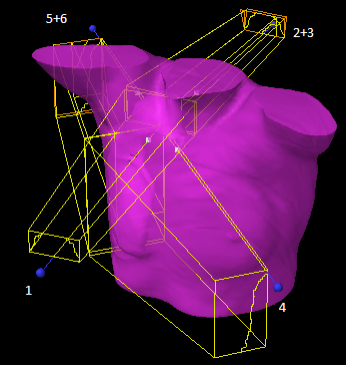
Illustration of the patient specific plan. Field numbers indicated in the figure. Fields 1 to 3 cover the cranial part of the target (lymph nodes); fields 4 to 6 are tangential fields covering the residual breast tissue.

The patient case selected as relevant to locoregional treatment in our clinic has a plan with six fields, as described in Table 1 and illustrated in Fig. 1. The Monte Carlo calculation of each of the four main fields, excluding MLC and wedges, is validated against measurements using a water box geometry and setting the gantry angles to zero. Furthermore, the combined dose distribution from the two main anterior fields (1 and 4) and the two posterior fields (2 and 5), respectively, is analyzed without MLC and wedges. The dose level for the fields, separate and combined anterior/posterior, is verified by ion chamber measurements, ($0.125\;{\text{cm}}^{3}$ PTW Semiflex chamber 31010; Freiburg, Germany), centrally in the field and in the tail region just outside the field at 3 cm depth in solid water. The shape of the separate field profiles is verified with the ion chamber profiler device IC Profiler (Sun Nuclear Corp., Melbourne, FL).

**Table 1:** Patient plan details for fields 1 to 6. Fields 1, 2 and 3 are anterior–posterior fields applied to the cranial part of the target (lymph nodes) for planning protocol with 1 mm overlap (Y1 = 0.1 cm). Fields 4, 5, and 6 are tangential fields covering the caudal part of the target (i.e., the breast tissue). All fields involve MLC

			*Lower (X) Jaw Position (cm)*		*Upper (Y) Position (cm)*			
*Field Number*	*Energy (MV)*	*Gantry Angle (°)*	*X1*	*X2*	*Y1*	*Y2*	*Wedge*	*MU*
1	6	10	5.5	5.7	0.1	5	No	145
2	15	183	5.7	5.5	0.1	5.3	$20^{\circ }$	70
3	15	183	3.5	5.5	0.1	5.3	No	11
4	6	50	11.5	0.5	20	0	No	114
5	6	229	0.5	11.5	19.5	0	$15^{\circ }$	106
6	6	229	0.5	6.9	14.7	0	No	11

Monte Carlo calculations for the tangential posterior field 5 is also verified with wedge included using IC Profiler, as well as one ion chamber measurement centrally in the field. The special consideration of field 5 is motivated by its asymmetry and length in combination with the wedge.

### B. Dose distribution in patient geometry

The dose distribution for the selected treatment plan is calculated with 0.15×0.15×0.15 cm resolution in the patient CT images from a Toshiba Aquilion LB CT (120 kV) (Tokyo, Japan). Tissue segmentation is performed by the formalism in Schneider et al.,^(10)^ as reported earlier.^(6)^ Clinical target volume (CTV) and planning target volume (PTV) are delineated according to clinical routine at our hospital.

The MC data are not converted to dose to water, wherefore dose to tissue is reported. The 3D dose distributions are imported as DICOM dose files via Vega library^(11)^ in the Eclipse v. 11.0 (Varian Medical Systems) TPS for viewing and DVH analysis, as well as acquiring of the dose‐volume‐parameters such as $D_{98\%}$ and $V_{95\%}$.

Dose distributions in the patient CT images are obtained for the following five cases of junction between the cranial fields and the tangential fields: 2 and 1 mm gap, a perfect match, as well as 2 and 3 mm overlap. The gap and overlap cases are simulated by shortening or extending the cranial fields. The dose‐volume parameters are evaluated for all cases.

Calculations are carried out on a Linux cluster at the National Supercomputer Centre Linköping, Sweden. The statistical uncertainty (one standard deviation (SD)) of the reported MC dose is about 2% in the target region.

## III. RESULTS

### A. Validation of MC beam calculations

**Figure A.1:**
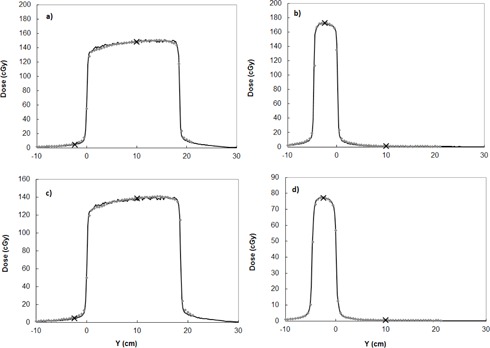
Dose profiles in water at 3 cm depth for the main fields of the validation plan: (a) tangential anterior field; (b) lymph nodes anterior field; (c) tangential posterior field; (d) lymph nodes posterior field. Black lines=MC calculations, gray dots=IC Profiler measurements, large crosses=ionization chamber measurements.

The difference between the ionization chamber measurements centrally in the field and MC data is 0.2%–0.9 % for the four separate main fields (MLC and wedges excluded) where the uncertainty of the MC data is negligible and the measurement error (95% significance level) is estimated to below 0.2% for all fields. The comparison between IC Profiler measurement and MC calculation is shown in Fig. A.1 in the Appendix, Ion chamber measurements are also shown in Fig. A.1. The agreement between MC and ionization chamber measurements for the combined anterior/posterior fields is evaluated in the same points as for the separate fields (i.e., two points in each summed dose distribution) and is within 1%.

**Figure A.2:**
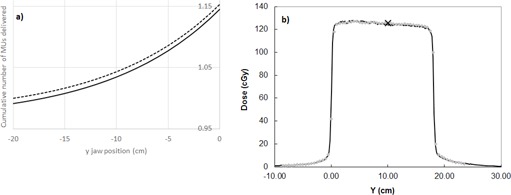
The STT for the wedge tangential posterior field (a). Backscatter corrected (dashed) shown together with the original uncorrected STT (solid) given by the manufacturer for wedge angle 15°. Validation profile (b) at 3 cm depth in water for the tangential wedge field (6 MV). MC calculations (solid black line) compared to IC Profiler measurement (grey dots) — a relative measurement normalized to the absolute ion chamber measurement (cross).

An example of the validation of the wedge field calculations is shown in Figure A.2(b) in the Appendix, where IC Profiler measurement are compared to MC data for the tangential posterior field, 5, including 15° wedge. The agreement between MC and measurement is for this case 0.9% centrally in the field.

The MLC component of the MC model is also found to produce results in good agreement with measurements. The results are not shown here since the MLC does not define the field edges in the junction region.

### B. Dose distribution in patient geometry

**Figure 2:**
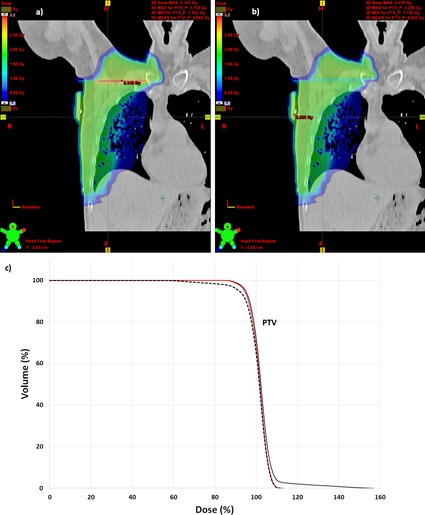
Illustration of the dose distributions in a plane 3 cm dorsal from isocenter for the situation of a) fields overlapping 3 mm and b) with a 2 mm gap. Color scale in Gy per fraction ranging from 0.2 to 3.16. DVH (c) for the case of 3 mm overlapping fields (solid line) and 2 mm gap (dashed line) between the fields covering the lymph nodes and the tangential fields irradiating the breast tissue. Perfectly aligned jaws shown for reference (red line).

The underdosage (2 mm gap) and overdosage (3 mm overlap) in the target volume is illustrated in the dose‐volume histogram (DVH) (Fig. 2). Plan evaluation parameters for PTV, body, and PTV‐body are listed in Table 2. $D_{98\%}$ and $D_{2\%}$ (near minimum and near maximum dose according to ICRU report 83^(12)^) are presented in the table to avoid point doses. PTV is $507.6\;{\text{cm}}^{3}$.

To further quantify the increased dose in the junction region in the case of field overlaps, the maximum width in craniocaudal direction of the volume covered by 110% isodose is estimated. The values obtained are 1.5 cm and 2.1 cm for 2 mm overlap and 3 mm overlap, respectively. The width of the volume covered by 120% isodose is 0.4 cm and 0.6 cm for 2 mm and 3 mm overlap, respectively. One hundred and ten percent (110%) and 120% isodoses are not observed in the case of perfect alignment of jaws.

The impact of jaw positioning errors on the lung dose is mainly due to the uncertainties of the cranial fields (when extended in caudal direction more lung is in‐field). Relevant subregion can be analyzed to further quantify the local effects that are barely seen in the DVHs. A subvolume of $145\;{\text{cm}}^{3}$ lung tissue around the junction region with 3 cm width in craniocaudal direction is considered. The mean dose changes from 23.8 Gy for perfectly aligned jaws (prescribed dose to the tumor is 50 Gy in 25 fractions) to 26.3 Gy for a 2 mm jaw positioning change (1 mm extension of field according to protocol and 1 mm uncertainty) — an increase of 2.5 Gy. Furthermore, the results in this study indicate hot spots in the lung tissue close to the chest wall in the case of field overlap. The dose level and volume of those hot spots are difficult to estimate because of interface uncertainties in the MC tissue segmentation procedure involving voxel interpretation of the CT scan. The lung tissue (defined by the clinical segmentation wizard) in the subvolume considered above has an increase in $D_{2\%}$ from 44.5 Gy (perfectly aligned jaws) to 45.4 Gy for a 2 mm overlap. The moderate increase in $D_{2\%}$ indicates that the volume of the hot spots is small.

**Table 2:** Plan evaluation measures for PTV, body, and body‐PTV

		*Jaws 2 mm Apart*	*Jaws 1 mm Apart*	*Jaws Perfectly Aligned*	*Fields Overlapping 2 mm*	*Fields Overlapping 3 mm*
PTV	$V_{95\%}(\%)$	90	91	94	95	95
	$V_{105\%}(\%)$	16	16	17	22	23
	$V_{110\%}(\%)$	0.2	0.3	0.4	3.0	4.0
	$V_{120\%}(\%)$	0.0	0.0	0.0	1.4	2.1
	$D_{2\%}(\%)$	108	108	109	113	121
	$D_{28\%}(\%)$	85	88	91	92	92
	$D_{\text{mean}}(\%)$	101	101	101	102	103
Body	$V_{105\%}({\text{cm}}^{3})$	207	216	224	268	283
	$V_{110\%}({\text{cm}}^{3})$	13	13	15	47	59
	$V_{120\%}({\text{cm}}^{3})$	0	0	0	15	25
Body‐PTV	$V_{95\%}({\text{cm}}^{3})$	503	510	526	547	558
	$V_{105\%}({\text{cm}}^{3})$	126	129	133	153	164
	$V_{110\%}({\text{cm}}^{3})$	11	11	12	31	37
	$V_{120\%}({\text{cm}}^{3})$	0	0	0	5	14

$V_{95\%}(\%)=90$ means that 90% of the organ volume received 95% of the prescribed dose or more; $D_{2\%}(\%)=108$ means that 2% of the organ volume received 108% of prescribed dose or more.

## IV. DISCUSSION

When gap is present, the largest concern is to evaluate possible cold spots in the target volume. The $D_{98\%}$ (near minimum dose) in the PTV is reduced from 91% for perfectly aligned fields to 88% and 85% for a 1 mm and 2 mm gap, respectively (see Table 2). The target coverage expressed as the PTV volume covered by the 95% isodose, $V_{95\%}$, is reduced from 94% to 91% and 90% respectively for a 1 and 2 mm gap. Thus, for 95% isodose coverage there is not a large distinction between gaps of 1 or 2 mm. When overlap is present, the PTV volume covered by 105% and 110% isodoses increases. A volume covered by 120% isodose appears, as well. However, when comparing the two cases of overlap, the largest effect is seen for $D_{2\%}$ (near maximum dose). This is to be expected, since the effect of overlapping fields is restricted to a small part of the dose distribution. In the clinical evaluation of a treatment plan, the risk for hot spots in target may not be the largest concern, but rather the risk for hot spots in normal tissue.

When overlap is present, even the volume outside target (Body – PTV in Table 2) covered by 110% isodose increases, from $12\;{\text{cm}}^{3}$ to $37\;{\text{cm}}^{3}$ and $37\;{\text{cm}}^{3}$ for 2 and 3 mm overlap. Also, a region of $15\;{\text{cm}}^{3}$ confined by 120% isodose appears for 2 mm overlap and increases to $25\;{\text{cm}}^{3}$ for 3 mm overlap. The region exposed by 110% dose or more does not include lung tissue, but other organs at risk, such as the plexus brachialis, may be present in this region. The dimensions of the 110% region in the craniocaudal direction are about 1.5–2 cm larger than in the case of perfectly aligned jaws. Thus, the everyday setup uncertainty we observe at our hospital cannot fully smoothen the effect.

The changes in mean dose, $V_{20\text{Gy}}$ and $D_{2\%}$ for the ipsilateral lung are small due to a large organ volume. However, the analysis of the 3 cm wide subvolume in the lung around the junction reveals larger changes, as pointed out in Results section B. Thus, analysis of small regions may be more appropriate to detect local dose changes than investigation of the dose distribution in whole lung. It should be stressed, that the definition of the subvolume is not based on anatomical features and the dose evaluation parameters have no clear clinical meaning.

**Figure A.3:**
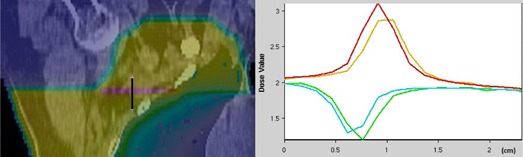
To the left the dose distribution for 3 mm overlapping fields is shown where the black line indicates the position of the dose profile shown in the graph to the right. MC dose profiles for 2 mm gap and 3 mm overlap (green and yellow) compared to AAA dose profiles (blue and red) for the same cases.

Analysis of the test calculations by AAA reveals a qualitative agreement with the MC results and conclusions. An example of the dose levels predicted in soft tissue in the junction region by AAA and MC, respectively, is shown in Fig. A.3 in the Appendix. Larger differences are observed between MC and AAA for profiles involving lung. More detailed quantitative comparison between AAA and MC data would require thorough investigation of AAA performance in penumbra regions and interfaces between soft tissue, bone, and lung tissue. The investigation of the impact of choice of algorithm in the clinical treatment planning system is important, but beyond the scope of this study.

The two treatment planning protocols, namely, planning without overlap of adjacent fields and planning with 1 mm overlap, can be discussed on the basis of the dose distribution analysis. For planning without overlap, the extreme cases are 2 mm gap and 2 mm overlap, respectively. The risk for insufficient coverage of PTV (e.g., decrease of $V_{95\%}$ by 4%) should be balanced with the risk for increased dose to healthy tissues (e.g., 110% dose to $47\;{\text{cm}}^{3}$ soft tissue) and increased local dose to the ipsilateral lung compared to the reference case with a perfect field alignment. For planning with 1 mm overlap, the extreme cases are to have 1 mm gap and 3 mm overlap of the adjacent fields. This strategy is used to secure the PTV coverage, but will entail an increased risk for higher doses to healthy tissues. Since $V_{95\%}$ is similar for both cases of gap, the advantage of the planning protocol with 1 mm overlap over this without overlap is not clearly seen. The risk for increased dose to soft tissue and lung are seen to be higher for 3 mm than for 2 mm overlap (and definitely higher than for perfectly aligned jaws). However, more detailed knowledge is needed on the risk for recurrence in the junction region and the clinical significance of the local increase of the lung dose before the effects can be properly evaluated. Risk factors like radiation induced brachial plexopathy, for example, should be taken into account, as well, and a maximum dose of 54 Gy (or lower to take into account the risk of increased dose due to uncertainties in jaw positioning) to plexus brachialis should be added to the analysis.^13^, ^14^ Elaboration on specific clinical recommendations might require different approaches, depending on treatment technique, diagnosis, and other patient specific circumstances, which is outside the scope of this study.

The results from this case study indicate that the use of a planning protocol with 1 mm overlap can be debated. Further studies on more patients are valuable to establish the dominantly negative effect found in this study of using a treatment protocol based on 1 mm field overlap.

In general, the policy of the treatment planning protocol depends on the rules for target delineation. According to the Swedish national guidelines, the remaining breast parenchyma and ipsilateral regional lymph nodes in the axillary level III and supraclavicular fossa are included in one CTV and, consequently, comprise one PTV. An alternative approach to target volume delineation is to consider the residual breast tissue and the lymph nodes as separate CTVs and consequently PTVs.^(14)^ In this way, different constrains can be defined for each PTV. In the work cited above, it is concluded that gaps between adjacent lymph node volumes should be avoided and the importance of a homogeneous dose in the intersection between different lymph node targets is stressed. Gaps between the residual breast tissue and the lymph nodes are not discussed. An interface between the residual breast tissue PTV and lymph node PTVs may allow larger flexibility in the planning stage.

Achieving a good PTV coverage has high priority in the treatment planning procedure. It is important to avoid gaps and, therefore, a planning protocol with 1 mm overlap was the choice at our hospital (i.e., for all locoregional breast cancer treatments). The results from this case study promote and facilitate a discussion on how the overlap can be adjusted for different groups of patients stratified, for example, according to stage of cancer. For the different groups of patients, avoiding underdosage of PTV and reducing dose to healthy tissues may have different priorities.

A variable placement of the junction between the adjacent fields may be implemented to smooth out the dose inhomogeneity in the junction region. The implementation may raise practical issues of having two active plans for a patient. Also the effects of the position of the junction on the dose coverage should be considered. This case is not investigated in the current work.

As the patient positioning techniques are improved by for instance daily imaging and surface scanning the setup errors are expected to decrease and no longer smoothen the effect of field gaps/overlaps to the same degree. Furthermore, less smearing due to setup errors is also true for the case of hypofractionation. Hypofractionation also means that the biological effect of hot spots will be larger.

## V. CONCLUSIONS

A treatment planning protocol with 1 mm overlap does not considerably improve the coverage of PTV in the case of erroneous jaw positions causing gap between fields, but increases the overdosage in PTV and the dose to healthy tissue, in the case of overlapping fields, for the case investigated. Therefore, a treatment planning protocol including 1 mm field overlap can be questioned. Before recommendations are made further investigations are needed, which should consider, for example, decreased daily setup errors, hypofractionation, and negative side effects in healthy tissue.

## ACKNOWLEDGMENTS

This study was supported by grants from the King Gustav V Jubilee Clinic Cancer Research Foundation, Lions Cancer Research Foundation, Assar Gabrielsson Research Foundation, and Percy Falk Research Foundation.

## Appendix A: The Monte Carlo Model

### A. Monte Carlo model, absolute dose calculations

The formalism for conversion of the MC dose in Gy per primary history to the dose in Gy for a certain number of monitor units MU (denoted further in the text as absolute dose) is based on simulations of the calibration geometry and corrections for the effect of backscattered radiation to the monitor chamber, as described in Popescue et al.^(A1)^ The accelerators in our clinic are calibrated in water at 10 cm depth at source‐to‐surface distance (SSD) 90 cm for a 10 cm×10 cm field. A backscatter correction factor (BSCF) is used that relates the amount of backscattered dose for a certain field to the calibration field size. A linear dependence is considered between the backscattered dose to the monitor chamber and the field size as suggested by Verhaegen et al.^(A2)^ It is assumed that the effect of the components located below the upper Y jaw, namely the lower X jaw and the MLC, is negligible. This assumption is consistent with the results reported on the dominating effect of the upper Y jaw on the backscatter compared to that of the lower X jaw.^16^, ^17^ The BSCF is therefore only dependent on the field length in the Y direction (FSy) and is given by:

(A1) $$BSCF(FSy)=\frac{a+b*10}{a+b*FSy}$$

New parameter values of *a* and *b* in Eq. (A1), specific for our accelerators, are obtained, namely, a=1.034(1.028) and b=−0.00085(−0.00070) for 6 (15) MV, respectively. The field sizes included in this optimization procedure are 4×4 cm, 20×20 cm, and 40×40 cm symmetrical square fields, as well as 4×20 cm and 20×4 cm symmetrical rectangular fields.

Wedge fields are generated by the DYNJAWS^9^, ^18^ code option following Varian Enhanced Dynamic Wedge (EDW) implementation. The dynamic movement of the upper jaws is controlled by the so‐called segmented treatment tables, STT. Each STT contains information on the jaw position versus dose delivery information at different instances of the EDW field in form of cumulative weighting of monitor units (MU). A single STT, (the one for 60° wedge), is used to generate all the other STTs for various field sizes and wedge angles. The input file for DYNJAWS is generated by the AUTOJAWS script.^(A5)^

For wedges, the backscatter correction is applied on the differential segmented treatment table; ${\text{STT}}_{\text{diff},i}={\text{STT}}_{i}–{\text{STT}}_{i-1}$, where i is an index indicating the row of the STT. To facilitate the writing in Eq. (A2), it is defined that ${\text{STT}}_{0}$ = 0. The row‐index, i, varies from 1 to maximum number of rows in the STT. Each row of the backscatter corrected STT, ${\text{STT}}_{\text{bscorr}}$, is thereby given by:

(A2) $$STT_{bs\text{co}rr,i}=\sum_{1}^{i}((STT_{i}-STT_{i-1})*BSCF(FSy_{i}))$$

In this way, the backscatter effect is taken into account when simulating the jaw movement. The backscatter corrected STT is normalized to the number of cumulative monitor units, delivered at the last position of the jaw, before it is used in the EGSnrc/BEAMnrc for producing a phase space. The number of MUs of a wedged field is in the treatment plan equal to the cumulative number of MUs delivered at the last position of the jaw. Therefore, a backscatter correction factor (denoted global in the text) is needed also for wedged fields so that the total number of MUs can be corrected in a similar way as for to the nonwedge fields. This global backscatter correction factor used for wedge fields in the conversion of the MC dose to absolute dose is obtained by the ratio between the cumulative number of MUs for backscatter corrected and non‐corrected STT, respectively.

### B. Monte Carlo model, validation

Validation results for the four main fields, no wedges included, are presented in Fig. A.1. The calculated anterior cranial and tangential fields 1 and 4, ((a) and (b) in Fig. A.1), and the posterior cranial and tangential fields, 2 and 5, ((c) and (d) in Fig. A.1), are compared with IC Profiler and point measurements by ionization chamber at 3 cm depth in solid water. The absolute dose (Gy for the specified number of MUs for each particular field) is given to test the validity of the dose conversion procedure. The agreement between MC and ionization chamber measurements in the central parts of the fields is within 1%. The MC calculations in the penumbra and the tail regions comply very well with the IC Profiler measurements as well as seen in the figure.

MC simulated wedge fields are validated by relative comparison of MC and measured dose profiles in solid water by IC Profiler (Sun Nuclear Corp.) containing 251 ion chambers with 2.9 mm width and 5 mm spacing, as well as by absolute dose measurements with ionization chamber CC13 (IBA Dosimetry, Schwarzenbruck, Germany). The validation of the backscatter correction for the wedged fields is shown for one of the patient treatment fields in Fig. A.2. The special consideration of this field is motivated by its asymmetry and length in combination with the wedge. Part of the MUs are delivered when jaw opening is exceptionally far away from the central axis (i.e., 19.5 cm), as compared to the small wedged posterior fields of 15 MV photons irradiating the cranial part of the target.

For simulated and measured wedge profiles for 6 MV and 15 MV, symmetric 20 × 20 cm field with 45° wedge an agreement within 1.6% is observed, except in the horns where deviations up to 3.6% are detected. For the MLC validation, the agreement between theoretical and experimental data is within 1.5%, except for the tails where the deviation is 2.5%.

### C. Dose profiles in junction region

An example of the dose levels predicted in the junction region by AAA and MC, respectively, is shown in Fig. A.3.
